# “Yeah, I Mean, You’re Going to Handball, so You Want to Use Balls as Much as Possible at Training”: End-Users’ Perspectives of Injury Prevention Training for Youth Handball Players

**DOI:** 10.3390/ijerph19063402

**Published:** 2022-03-14

**Authors:** Karin Moesch, Sofia Bunke, Jennie Linnéll, Eva M. Brodin, Alex Donaldson, Eva Ageberg

**Affiliations:** 1 Department of Psychology, Lund University, 22100 Lund, Sweden; sofia.bunke@psy.lu.se; 2 Department of Health Sciences, Lund University, 22100 Lund, Sweden; jennie.linnell@med.lu.se (J.L.); eva.ageberg@med.lu.se (E.A.); 3 Department of Educational Sciences, Lund University, 22100 Lund, Sweden; eva.brodin@ahu.lu.se; 4 Centre for Higher and Adult Education (CHAE), Department of Curriculum Studies, Faculty of Education, Stellenbosch University, Stellenbosch 7600, South Africa; 5 Centre for Sport and Social Impact (CSSI), La Trobe University, Bundoora, VIC 3086, Australia; a.donaldson@latrobe.edu.au

**Keywords:** injury prevention program, adolescents, handball, end-user involvement

## Abstract

Young handball players experience high injury rates. Specific injury prevention programs reduce injury rates but are not well implemented into youth players’ training. The ‘Implementing injury Prevention training ROutines in TEams and Clubs in youth Team handball (I-PROTECT)’ project addresses this challenge. The aim of this study was to investigate how youth handball coaches and players experienced the recently developed I-PROTECT GO pilot program, by focusing on barriers and facilitators. Three focus group interviews were conducted with coaches and players, and their answers were analyzed using a general inductive approach. The participants appreciated the program and found it useful for their context. The participants’ statements about facilitators and barriers centered around the categories of resources, exercises, program design, and learning. Facilitators that emerged were motivating exercises (e.g., handball-specific), a helpful set-up (e.g., variation), having a clear purpose of exercises, the possibility to fulfil basic psychological needs while training, receiving instructions and feedback, and having role models. Barriers that emerged were limited space and material, difficulties with exercises, an unhelpful set-up (e.g., too repetitive), and undisciplined training. It is important to address perceived barriers and facilitators among coaches and players when developing injury prevention training programs to enhance the uptake of such training.

## 1. Introduction

Young athletes, particularly young handball players, experience high injury rates [1,2]. Sustaining an injury results in physical harm, can also cause psychological suffering, and is a risk factor for developing mental health problems [3,4]. Meta-analyses and systematic reviews show that specific injury prevention programs reduce the overall injury rate among adolescent athletes in team sports by approximately 40% [5,6,7], and such interventions in youth handball show promising results for reducing lower limb injury [8,9].

However, there are challenges in implementing and sustaining the use of injury prevention training [10]. DiStefano et al. [11] identified a gap between short-term improvements (mainly occurring in controlled environments, i.e., during a study) and long-term implementation strategies (mainly occurring in uncontrolled environments). Studies confirmed that, in uncontrolled environments within youth team sport athletes, complete injury prevention programs are rarely used, and single injury prevention exercises are only used to a moderate extent [12,13]. Even in a controlled environment, low utilization rates of injury prevention exercises in youth floorball players were reported [14].

Several barriers have been identified that may help increase our understanding of the lack of use of injury prevention training. Players, coaches, and/or physical and medical staff can find the injury prevention exercises boring and/or monotonous [12,13,15,16,17], too time consuming [12,15,16,17,18], and/or lacking sport- and age-specificity [12,15,19]. The lack of player motivation to perform injury prevention training is also a major barrier for sustained implementation [13,16,17,18]. 

Conversely, exercises that are fun, game-like, sport-specific, challenging, and that include progression and variation have emerged as facilitators for sustained implementation of injury prevention training [12,13,16,17,20]. This may be because exercises with these characteristics are more likely to generate positive consequences for the end-user (e.g., by inducing positive emotions), which is considered a powerful tool to maintain or increase the frequency of a behaviour over time, through a process known as reinforcement [21]. To increase the chance that end-users experience positive consequences when executing injury prevention training, it is important to include their perspective when developing the intervention. Including end-users has emerged as an important consideration when developing injury prevention training [22], particularly to establish context-specificity [10].

We initiated the ‘Implementing injury Prevention training ROutines in TEams and Clubs in youth Team handball (I-PROTECT)’ project to address the challenge of the low utilization rate of injury prevention training, with the aim to achieve adoption, high-fidelity use, and maintenance of evidence-based and handball-specific injury prevention training in youth players. The project has an interdisciplinary and holistic approach, with a focus on end-user engagement throughout the project [20,23]. In a recent study [24], researchers/handball experts and end-users co-created a first pilot version of injury prevention exercises including physical principles and psychological aspects (i.e., motivation using principles from self-determination theory, task focus, and body awareness) to be integrated within handball practice. The program development was based on the Health Action Process Approach (HAPA) [25] behavior change theory and self-determination theory (SDT) [26]. The integration of these theories into the program is described in more detail in Ageberg et al. [23] for HAPA and in Ageberg et al. [24] for SDT. The overall purpose of this study was to investigate how youth handball coaches and players experience this pilot version (the I-PROTECT GO pilot program) for injury prevention training, by focusing on barriers and facilitators.

## 2. Materials and Methods

### 2.1. Design

Given the aim of the study, we decided to use a qualitative approach in the form of focus group interviews. Focus groups are a resource-friendly way to elicit opinions and views on a focused topic (i.e., the I-PROTECT GO pilot program) in a small group of persons having the same experience with the specific topic, enabling interaction and exchange within the group [27]. The Regional Ethical Review Board in Lund approved the I-PROTECT project (EPN 2014/713). All participants received the participant information and provided informed consent to participate in the study.

### 2.2. Intervention

Five teams (two female, three male), including six coaches (one female, five males) and 127 players, from the two handball clubs in Southern Sweden participating in the I-PROTECT project, took part in the four-week study during September 2019. The teams were recruited through the coaches who participated in workshops to test and evaluate earlier versions of handball-specific injury prevention exercises and volunteered to take part in the present study. Three of the authors (SB, JL, EA) conducted a workshop before the study, in which JL educated the coaches about the exercises and the use of the I-PROTECT GO pilot program. JL also visited all teams once during the intervention period to answer any question or concerns about implementing the program. Each coach received a tablet with the I-PROTECT GO pilot program installed before the study started.

The I-PROTECT GO pilot program was delivered as a pre-defined program, as previously requested by coaches [24]. The program included exercises focused on physical (movement techniques for upper and lower limbs, respectively, and muscle strength) and psychological aspects (increase end-user motivation, task focus, and body awareness). The exercises were integrated within warm-up and handball skills training [24]. In the present study, six exercises were integrated during warm-up, and eight exercises were integrated during handball skills training. Four exercises had either two or three levels of difficulty for variation. All teams had handball practice three times per week; therefore, the handball-specific injury prevention exercises were distributed across three training sessions. The warm-up exercises were included in all training sessions, along with either upper limb, lower limb, or muscle strength exercises during handball skills training (Table 1). The coaches were asked to perform all three, and a minimum of two, sessions each week, during the 4-week intervention. For a more thorough description of the program and its exercises, we refer the interested reader to Ageberg et al. [24].

### 2.3. Focus Group Interviews

#### 2.3.1. Participants

All coaches who participated in the intervention were contacted and asked to participate in the coach focus group interview. Furthermore, all players were informed that they could participate in a player focus group. A distribution of age and gender guided the final sample within the following groups: (i) “young players”: a group of six players aged 14–15 years (two females, four males); (ii) “older players”: a group of six players aged 16 years (three females, three males); and (iii) “coaches”: a group of five coaches representing all five teams (one female, four males). All three groups included individuals from both clubs.

#### 2.3.2. Data Collection and Analysis

The focus group interviews were held shortly after the intervention was completed at the main location of both clubs. They were led by two of the authors (KM and JL). Upon arrival, participants were informed about the topic and the process of focus group interviews. Based on an interview guide, the participants were asked to share their experiences of (i) the exercises, (ii) the set-up of the four-weeks intervention, and (iii) the mobile application I-PROTECT GO (for coaches only). Follow-up questions were used to encourage participants to expand on responses and to gather participants’ experiences of facilitating and hindering factors for sustained injury prevention training. The focus group interviews lasted on average 55 min (range 46–65 min), were video recorded, and were transcribed verbatim using an external service provider. Participants were deidentified in the interview transcripts. The first author (KM) checked the transcripts for accuracy against the video recordings and corrected any anomalies. The transcripts were imported into NVivo 12. Data analysis was guided by the principles of the general inductive approach [28]. The first author (KM) proceeded through the steps (i) thorough reading of the text, (ii) creation of categories, and, finally, (iii) revision and refinement of categories to result in a final version [28]. Three co-authors (SB, EMB, EA) acted as critical friends, overseeing the process done during the above-mentioned steps.

## 3. Results

In Table 2, overarching categories and end-users’ evaluation of facilitators and barriers for injury prevention training are displayed. *Resources* refer to external factors in the environment that affect the execution of injury prevention training. Statements related to different aspects of the program exercises are categorized into the theme *exercises*. The theme *program design* includes how the program was composed and delivered. Finally, the theme *learning* refers to factors in the learning environment that can hinder or facilitate implementing injury prevention training.

### 3.1. Resources

**Role models.** The participants stated that they found it a motivating resource to have a well-known coach coming for a visit and working with I-PROTECT. Likewise, the participants suggested that it could be inspiring to have role models (i.e., other handball players) advocating for or performing the program. A coach stated: “…It was ‘wow, they do it’. So you see that the more senior players actually do it”.

**Limited space and material.** Limited space, for example, when the injury prevention training was done before having access to the sports center (i.e., in the corridors), can become a problem. Not having enough resources (e.g., elastic bands) for all players was also a factor that decreased the likelihood that players would perform the exercises. The coaches highlighted that they do not always train at the same sports center, so the program resources needed to be easy to transport to other venues.

### 3.2. Exercises

**Motivating exercises.** The participants expressed that handball-related exercises (e.g., exercises including a handball and exercises including handball-specific situations and/or movements) were motivating. One female player expressed this as follows: “Yeah, I mean, you’re going to handball, so you want to use balls as much as possible at training”. Likewise, having a competitive element in the exercises was considered motivating, as a male player stated: “Kind of everything with a competitive touch gets more fun”. The participants also highlighted that they liked that the program included exercises involving the whole body, focusing on all body parts that are central in handball (i.e., lower and upper extremities and core). Doing exercises with a teammate was also motivating, as a female player stated: “I liked the exercises that you performed with a teammate. It gets a bit more fun like that”. Another motivating point was when exercises had several levels of difficulty, so players could progress to more difficult exercises once they completed the easier ones. A female player stated that “They were good exercises in that way that you always could develop them. That there was kind of another level”.

**Difficulties with exercises.** Although the participants generally liked the exercises that were performed in pairs, they also expressed concern that it could be difficult to find a teammate (i.e., when the team has an unequal number of players) and that at times, the pairs were not on the same level, which could lead to players not performing the exercise correctly. A coach stated it like this: “The problem with this is sometimes also that they end up with someone who actually is stronger or weaker than themselves”. The participants also reported that it was difficult to understand how to do some exercises, which hinders motivation to do them and decreases the odds that they will be executed correctly. The participants also reported that one exercise (“plank on knees”) was painful on the kneecap.

### 3.3. Program Design

**Helpful set-up.** The participants highlighted that injury prevention training should be completed in a reasonable time-period, and the consensus among the participants was around 15 min per training. Related to that, the participants also pointed out that it was important to have more time available for each exercise initially (i.e., when learning the exercise) and that more exercises could be added once they got more familiar with them. A female player expressed it as follows: “But it felt better once you had learned the exercise. Then it was easier to have more exercises but that there maybe were too many in the beginning. So that you escalate it, kind of.” Establishing a habit was considered an important factor for making injury prevention training sustainable over time. A male player said: “We knew that every practice started with this stop, three-step-stop, so we knew we did it. So, we kind of got used to it, so we knew what to do. This was quite good”. Another motivating factor was variation, both in terms of selecting single exercises as well as the combination of exercises in the program. A statement from a female player describes this: “Injury-prevention training is maybe not always very much fun, but by varying it, it can become something fun”.

**Clear purpose of exercises.** Understanding how an exercise decreased risk of injury and enhanced performance was considered motivating. As a player stated: “Yes, the purpose of the exercises, so you understand why you do them”. A coach described the performance enhancement purpose as follows: “The exercises that are directly related to ‘if you do this you will shoot better’, then they get hungry on it [the program]”.

**Unhelpful set-up.** The participants stated that a set-up that was too repetitive in the sense that they always did the same exercises and combinations at every training hindered their motivation. In addition, a set-up that took too long was not helpful for participants´ motivation, as a female player stated: “It takes too long of a time from our practice”.

### 3.4. Learning

**Being educated.** Participants stated that being properly educated, optimally with access to multiple sources (e.g., verbal instruction from coaches, looking at videos, and/or reading in the app), was a facilitating factor for executing the exercises. Moreover, getting feedback from the coaches was considered motivating and important for correct execution. A female player described it as follows: “All coaches were there and observed so everyone did it correctly, and then you also got some feedback at the same time”.

**Self-determination.** The ability to choose exercises and/or levels of progression was considered motivating. The participants also raised the fact that it could increase motivation if one felt ownership of the program, which was meant in terms of having access to all information (both coaches and athletes) to enable participants´ own engagement in the training process. Another factor that was considered motivating was when players experienced improvement over time. A male player expressed this point as follows: “I´ve had problems with my shoulders before, and these exercises we did, I think they are quite good. It gets easier with them after some time. And you felt a little stronger already after some weeks.”.

**Undisciplined learning.** Some of the participants had experienced instances of undisciplined learning. For example, chatting with peers was one such instance, which clearly hindered the possibility to (correctly) perform the prescribed exercises. A female player described this as follows: “We were chatting too much. Because it easily gets like that when you´re doing injury prevention training, then it is, sort of, time for chatting”. Another example was that the players did not place full attention on the injury prevention exercises, which resulted in less repetitions and/or lower quality in execution. A coach stated this as follows: “They don´t have focus on which muscles are working”.

## 4. Discussion

This study qualitatively investigated coaches’ and players’ experiences about using the I-PROTECT GO pilot program (a program that was co-created by researchers/handball experts and end-users [24]) during their practices. The results show that the participants appreciated the program and that it was usable in their context. The participants’ answers revealed facilitators and barriers in relation to injury prevention training, which centered around the four categories of resources, exercises, program design, and learning.

Although the importance of involving end-users in developing and evaluating injury prevention programs has been promoted [10,23,29,30], injury prevention training programs have frequently been developed by researchers with little [9,31] or no involvement of end-users [32,33,34]. One recent exception is Bruder et al. [22], who involved experts and end-users when developing a context-specific program for female Australian Football players. They acknowledged though that their program was developed rapidly and that the involvement of experts and end-users was occasionally impeded during the process. The I-PROTECT GO pilot program is therefore unique, as it is co-created by researchers/handball experts and youth handball players and coaches using a cyclical development process over several years [24]. This resulted in a program that is genuinely handball-specific compared with existing programs with low sport-specificity [32], that incorporates a whole-body approach compared with programs solely focusing on upper or lower extremities [9,31], and that includes exercises for both warm-up and handball skills training compared with programs solely including warm-up and/or strength exercises [29,30,31]. Further, the I-PROTECT GO pilot program is the first injury prevention program incorporating psychological aspects in the intervention. Although several authors have highlighted the importance of psychological factors for sustained use of injury prevention training [16,18], no other training programs have incorporated psychological factors.

The feedback from participants indicates that incorporating principles from SDT [26] in the program, i.e., the ability to fulfil the three basic psychological needs of relatedness, feeling competent, and autonomy, was considered positive. The participants highly appreciated the exercises they could do with a teammate, which provided an opportunity to feel related with others. It was also considered motivating to see progress over the 4-week intervention, which induced a feeling of competence. Also related to an increased feeling of competence was that the participants found it motivating to get thorough instructions for new exercises and feedback from coaches to facilitate mastery of the exercises. Finally, the participants expressed that a facilitating factor was having a feeling of autonomy and ownership of the program. The ability to choose a specific level of difficulty for an exercise (as a player) or modify an existing program (as a coach; to increase autonomy), as well as incorporating more difficulty levels (to increase feeling of competence), are therefore recommended strategies for injury prevention training. These strategies were implemented in the further development of the I-PROTECT programs.

The results indicate that the participants’ experiences of the set-up in the I-PROTECT GO pilot program differed. While some participants found the intervention too time consuming and repetitive, other participants found the set-up reasonable in duration and appreciated variation in the exercises in combination with the habit that was built-up during the intervention period. A possible explanation for these different views is that not all coaches used the program as intended (i.e., to integrate some of the exercises into warm-up and some into handball skills training) but put the exercises in one block separate from other parts of practice, which could be experienced by the players as “it takes too long of a time from our practice” (statement of a female player). It emerged from earlier steps in developing the I-PROTECT GO pilot program [20,23] and was recently confirmed by Møller and colleagues [12] that injury prevention training needs to be integrated into regular practice. A take-away based on this result is that coaches need to be better educated about how to optimally integrate injury prevention training into their regular routine. For the I-PROTECT project, this will now be included in the coach education, in which it will be emphasized that the I-PROTECT idea is handball exercises including injury preventive principles and explained thoroughly how and when to integrate the exercises during a regular practice. Another take-away from this result is to slightly shorten future example programs. Participants stated that around 15 min of injury prevention training was feasible, which, though, optimally would be integrated into regular practice. This is in line with a meta-analysis that concluded that bouts of 10–15 min of injury prevention training appear sufficient to achieve a substantial injury risk reduction if done two to three times a week and over a prolonged period (i.e., 6 months) [35]. As such, both from an end-user perspective and an injury risk reduction point of view, injury prevention training of around 10–15 min per session seems to be feasible and sufficient.

Although the participants enjoyed exercises with teammates and exercises with a competitive element, the results also revealed some obstacles. These exercises introduce a risk that players will not focus fully on exercise execution but on winning the competition or on chatting with the teammate. From a practical point of view, teammate exercises require an even number of players in practice and pairs that are physically similar. Finding a good balance between leveraging the motivating effect of teammate and competitive exercises while still ensuring correct execution and task focus is an important challenge to tackle when developing injury prevention programs.

Beyond the new insights described above, some of our results on facilitators and barriers support earlier research. For example, having exercises that suit the context of youth handball emerged as a crucial facilitator in the present study. Participants highly appreciated that the exercises were handball-related (i.e., that the exercises included a ball, handball-specific skills, a teammate, and/or a competitive element) and that they included all body parts that are fundamental to handball. The in-depth involvement of end-users in developing the pilot program [24] might explain this result, and end-users will continue to be involved in the ongoing development of the program. The importance of having sport-specific programs has been highlighted earlier [12,16]. In line with previous research [13,16,36], variation emerged as a facilitator in the present study. Based on this result, the program will be expanded with more exercises, and coaches will have the opportunity to follow a predefined program that will be modified regularly. They will also be able to choose to design their own program from the available exercises. This step will hopefully further increase variation and, importantly, provide the opportunity for self-determined choices. Understanding the injury prevention and/or performance enhancement purpose of each exercise emerged as another facilitating factor from our study and is in line with earlier research [12,20,36]. Lastly, in line with previous research [12,19], our results confirm that it is motivating for participants to see other handball players and coaches who perform or advocate the program. The importance of role models in injury prevention training has been raised elsewhere [37].

Exercises that are difficult to understand, for example, when the focus is on activating small muscle groups, were considered barriers by the participants, which is in line with earlier research [38]. To enhance motivation and ensure correct execution, it is important to provide a comprehensive introduction to the exercises, for coaches to deliver feedback, and to ensure that there is enough time to learn the exercises correctly. Another barrier that emerged from the results was limited space and the need for specific resources, which has been reported in other studies [17,38]. Having a program with limited and easily accessible and/or portable equipment and the ability to perform parts of the program in limited space seems to be important to facilitate the implementation of injury prevention exercises.

### Strengths and Limitations

A strength of the present study is that both coaches’ and players’ experiences of implementing the intervention were explored. As players are the end-user group executing the exercises, it is crucial to understand their perceptions. In contrast, several earlier studies have solely investigated the staff’s perceptions about an intervention [13,15,19,39]. Another strength of our study is that the intervention was tested in the real-world setting to gather insights about its acceptability and usability to inform further development. Earlier research has mostly investigated general perceptions about facilitators and barriers of injury prevention training programs without evaluating a program that was implemented in the specific context [13,16,19,39]. The use of a qualitative design further allowed for an in-depth exploration of participants’ experiences and opinions of the intervention. Several similar studies used questionnaires to investigate participants’ perceptions [13,17,19], which does not provide the same depth of understanding. In support of our design, the use of qualitative methods has been promoted by researchers for this area [40,41].

This study has some limitations. First, the intervention was only implemented for four weeks, which impeded the possibility to assess the *sustainability* of injury prevention training. Second, we did not assess whether or how much the participants had experienced any injuries. Athletes who have sustained an injury during the current seasons are more likely to engage in injury prevention training [42], which also raises the hypothesis that one’s perception and experience of injury prevention training, as well as potential facilitators and barriers, may depend upon such personal experiences. A third potential limitation is that only three focus groups were conducted. One coach from each team but only 11 players (from a total of 127 players in the five teams) took part in the focus group interviews. However, there was a repetition of themes across all groups, suggesting that saturation was reached, and it is therefore assumed that the findings of the study may not have been different if more players had participated. Lastly, even though the real-life setting was considered a strength of the study, this design also involves a limitation as it was not possible due to practical reasons and was outside the scope of this study to assess program fidelity. This could be a valuable extension for future research.

## 5. Conclusions

In general, the participants appreciated the I-PROTECT GO pilot version and found it useful for their context. In particular, sport-specificity emerged as an important facilitator: handling a ball, practicing handball-specific skills, being with teammates, and a competitive touch, that is what the young handball players signed up for when joining a handball club, and these factors need to be transferred into injury prevention training to enhance sustained use. Other facilitators included having role models, a helpful set-up with, for example, exercise variation, having a clear purpose of “why” an exercise should be done, the possibility to fulfil basic psychological needs while training, and receiving clear instructions and feedback. A training program that fulfills these needs is more likely to generate positive consequences when training, which, in turn, increases the odds for sustained use. Perceived barriers included limited space and material, difficulties with exercises, a set-up that was too repetitive and time-consuming, and distractions in attention. Involving end-users when designing injury prevention training is a crucial step to reach sport-specificity and is strongly recommended for future injury prevention training program development.

## Figures and Tables

**Table 1:** Handball-specific injury prevention exercises integrated within warm-up and handball skills training, distributed across three training sessions per week during the 4-week intervention period.

Exercises		Training Session
**Exercises Integrated Within Warm-Up**		
**Exercise description**	**Aim of exercise**	
Plank with arm wrestling and ball	Core and grip strength, pairwise, competition. Two levels of difficulty	1, 2, 3
Single-leg standing balance with ball	Leg and grip strength, pairwise, competition	1, 2, 3
Running with foot plant *	Leg strength, mindful muscle activation, peer-feedback (optional)	1, 2, 3
Bow and arrow with resistance band *	Strengthen posterior part of shoulder, pairwise	1, 2, 3
Shoulder external rotation (upper arm close to body) with resistance band	Strengthen posterior part of shoulder, pairwise	1, 2, 3
Squat with partner	Leg and core strength, pairwise. Two levels of difficulty	1, 2, 3
**Exercises integrated within handball skills training**		
**Exercise description**	**Aim of exercise**	
Backwards throw *	Train posterior part of shoulder, wrist, pairwise, peer-feedback	1
Throw behind back	Train posterior part of shoulder, wrist, pairwise, peer-feedback	1
Overhead-throw	Train posterior part of shoulder, wrist, pairwise, peer-feedback	1
Integrated strength in technical skills training	Group exercise in which players choose exercises from circuit program. Strength exercises coupled with specific handball skills practice, i.e., shoulder: throwing; core: defence; jumping and landing: feint and cutting movements	2
Slow-motion feint with elevated arm	Leg strength, pairwise, mindful muscle activation. Three levels of difficulty	3
Jumping and cutting exercise	Leg strength, mindful muscle activation. Three levels of difficulty	3
Jumping and cutting exercise with catch and throw (level 1)	Leg strength, pairwise, mindful muscle activation	3
Jumping and cutting exercise with catch and throw (level 2)	Leg strength, pairwise, mindful muscle activation	3

Balance, coordination, posture, and flexibility were included in most exercises and are not specifically detailed in the aim of the exercise. * exercises from Fit to play (fittoplay.org).

**Table 2:** Overarching categories and end-users’ evaluation of facilitators and barriers for injury prevention training.

Facilitators	Category	Barriers
**Role models** Famous person coaching Successful players using/talking about program	RESOURCES	**Limited space and material** Limited or no access to material Occasionally restricted space
**Motivating exercises** Exercises with a teammate Competitive exercises Handball-related (with ball) Including whole body Exercises with increasing difficulty	EXERCISES	**Difficulties with exercises** Challenge finding a teammate Hard to understand how to do some exercises Painful to do one exercise
**Helpful set-up** Reasonable time demand and enough time per exercise Establishing a habit Variation in exercises and combinations **Clear purpose of exercises** Relationship to handball performance Relationship to injury prevention	PROGRAM DESIGN	**Unhelpful set-up** Too repetitive Too time-consuming
**Being educated** Receiving instructions from different sources and feedback Self-determination Choosing exercise/level: ownership Experiencing improvement over time	LEARNING	**Undisciplined training** Not paying attention: chatting with peers

## Data Availability

Data is available on reasonable request.
